# Novel electro-oxidation unit for electro-disinfection of *E. coli* and some waterborne pathogens during wastewater treatment: batch and continuous experiments

**DOI:** 10.1038/s41598-022-20451-w

**Published:** 2022-09-30

**Authors:** Mohamed S. Hellal, Bahaa A. Hemdan, Marwa Youssef, Gamila E. El-Taweel, Enas M. Abou Taleb

**Affiliations:** Water Pollution Research Department, National Research Centre, 33 El-Bohouth St., Dokki, Giza, 12622 Egypt

**Keywords:** Environmental sciences, Chemistry

## Abstract

The prime objective of the current investigation is to evaluate a promising alternative method for disinfection wastewater using a novel electro-oxidation unit. The study focused on determining the best-operating conditions from a techno-economic point of view to be applied to continuous flow simulating actual disinfection modules. The treatment unit consisted of a Plexiglas container with a 3 L volume containing nine cylindrical shape electrodes (6 graphite as anode and 3 stainless steel as a cathode) connected to a variable DC power supply. Determination of the best operating parameters was investigated in batch mode on synthetic wastewater by studying the effect of contact time, current density (CD), total dissolved solids concentration (TDS), and bacterial density. Moreover, the continuous mode experiment was considered on real wastewater from an agricultural drain and the secondary wastewater treatment plant effluent before chlorination. The batch mode results revealed that the best applicable operational conditions that achieved the complete removal *of E. coli* were at a contact time of less than 5 min, TDS of 2000 mg/L, and CD of 4 mA/cm^2^. Application of these conditions on the continuous mode experiment indicated the complete removal of all bacterial indicators after 5 min in the drainage wastewater and after 3 min in the secondary treated wastewater. Physico-chemical characterization also suggested that no chlorine by-products displaying the hydroxide ion formed due to water electrolysis is the main reason for prohibiting the growth of pathogenic microbes. The electrical consumption was calculated in the continuous mode and found to be 0.5 kWh/m^3^ with an operational cost of about 0.06 $/m^3^, including the cost of adding chemicals to increase the TDS. The results proved that this novel electro-oxidation unit is a robust and affordable disinfection method for complete bacterial removal from wastewater and is more environmentally benign than other conventional disinfection methods.

## Introduction

Inconsistencies between water availability and demand are increasing worldwide, especially in countries suffering from water scarcity like Egypt. By the year 2050, urban areas in these countries will be susceptible to water scarcity due to an increase of 80% in urban water demand due to rapid population^1^. To close the gap between water demand and supply, the reliance should be on alternative water sources such as reusing treated municipal wastewater. Tertiary or advanced treatment is necessary for urban reuse of municipal wastewater^2^. However, disinfection of this treated wastewater is mandatory to safely reuse as residual microbial pollutants such as *Escherichia coli* still exist in the treated wastewater. Disinfection is unquestionably crucial in water treatment to yield safe water free from these pathogenic microorganisms. Classification of water disinfection methods can be divided into three broad groups: chemical, physical, and physicochemical. Essential physical disinfection depends mainly on physical processes without any chemical that does not involve chemicals and relies on physical means without adding a chemical that may change water properties^3^. These methods varied between thermal disinfection (heating/boiling), ultra-sonication, membrane filtration, and UV irradiation^4^. However, this disinfection method has several disadvantages, such as the need for a long time, many personnel to monitor flushing times and temperatures of the water tanks, and only being effective for a short term^5^. Another type of disinfection is the chemical method that uses chemical compounds to deactivate waterborne pathogens in water and wastewater. The typical chemical disinfectants utilized in wastewater are sodium hypochlorite, chlorine dioxide, hydrogen peroxide ozone, (mono) chloramine, and chlorine gas, the most used^6^. Using chemicals in chemical disinfection have some inherent drawbacks to human health and to the environment, such as chlorinated by-products (DBPs), besides the high price of chemicals and high energy consumption^7^. Physiochemical disinfection includes chemical and physical approaches such as electro-disinfection, in which mixed oxidants (disinfection agents) are formed from electrolytic cells^8^. Recently there has been a great tendency to use electro-disinfection as an essential alternative technic to chemical disinfection for upcoming years^9^. This new technology can achieve an acceptable level of disinfection for treated wastewater with limited chemical additions in a short time.

The mechanism of electro-disinfection is based on the destruction of microorganisms by passing an electric current through the water using appropriate electrodes. Pathogenic bacteria are destroyed by various oxidants that are produced during the electrolysis of water^10^. There are two categories of electrochemical disinfection devices; electrolyzers, which are based on the direct interaction with contaminated water, and mixed oxidant generators, which use a concentrated brine solution to generate oxidizing species (e.g. free chlorine [Cl_2_], chlorine dioxide [ClO_2_], ozone [O_3_], hydrogen peroxide [H_2_O_2_], and other short-lived radicals) that can enhance the overall disinfection efficiency^11,12^. In free chloride waters, the oxidation of water on the anode surface produces physisorbed hydroxyl radicals M (^⋅^OH). At a limited release rate, ^⋅^OH reacts non-selectively with waterborne pathogenic agents or with a wide range of recalcitrant organics^9^. When the chloride is present, whether it is naturally present (e.g., toilet, wastewater, seawater) or is added artificially, reactive chlorine species (RCS) such as free chlorine ([Cl_2_], [HOCl], [ClO^−^]) and chlorine radical species ([^⋅^Cl_2_^−^], [^⋅^Cl]) are generated and considered as primary disinfectants^11^. In wastewaters, the organic matter could be oxidized in the electrolytic cell through two mechanisms: (i) Direct oxidation via a direct electron transfer to the anode, and (ii) mediated oxidation via the electro-generation of oxidizing species from wastewater itself or supporting electrolyte oxidation at the anode at high intensity^9^.

The types of electrochemical cells used in disinfection varied between electro-oxidation, electro-coagulation, and electrolysis. Electro-oxidation is the most functional unit as it is non-consumable, can generate oxidants without less chemical addition, and disinfection can occur directly through electron transfer to the anode^13^. Elect-disinfection degree factors are cell design, electrode types and shapes, time, electrolyte conductivity, applied current density, and pathogen concentration. The type and composition of the anode is a significant factor that plays an essential role in the efficiency of the electrochemical disinfection process because it affects the generation of oxidants and determines their nature and oxidation power. Previous studies have used various electrodes such as platinum, titanium, stainless steel, graphite, PbO_2_, boron-doped diamond (BDD), carbon-cloth, graphite and silicone rubber, and others^12,14^. Other operational parameters, such as current intensity, cell voltage, reaction time, pH, and temperature, are also crucial in optimizing the electrochemical disinfection process. Lui et al.^15^ studied the inactivation of *E. coli* in wastewater using a 140 mL electrochemical disinfection cell equipped with molybdenum carbide electrodes. They focused on the effect of electrode material and time on the disinfection of *E. coli,* and results revealed that 30 min and 2.0 V applied potential. Another study by Huang et al.^11^ investigated the disinfection of toilet wastewater using a 250 mL electrolysis unit containing a bismuth-doped TiO_2_ anode and stainless steel as the cathode.

The studies included a comparison between conventional chlorination methods and electro-disinfection methods. They revealed that 4 V potential and 60 min is sufficient for disinfection without generation of chlorine by-products. Although the previous studies investigated the parameters related to the electro-disinfection methods, few studies focused on optimizing these factors for actual application in a treatment plant, considering the availability of materials for construction and the operational cost. Consequently, the main aim of this analysis is to implement a revolutionary, affordable electro-oxidation system to evaluate operational parameters for the electro-disinfection of municipal wastewater. Contact time, current density, NaCl concentrations, and bacterial populations are deemed the considerable factors to be inspected. All preferred conditions for the operating system were engaged for the better inactivation effect of bacterial populations in both actual wastewater and synthetic contaminated water. Furthermore, the operational cost analysis in terms of electrical consumption was investigated.

## Materials and methods

### Electro-oxidation reactor design

**Figure 1:**
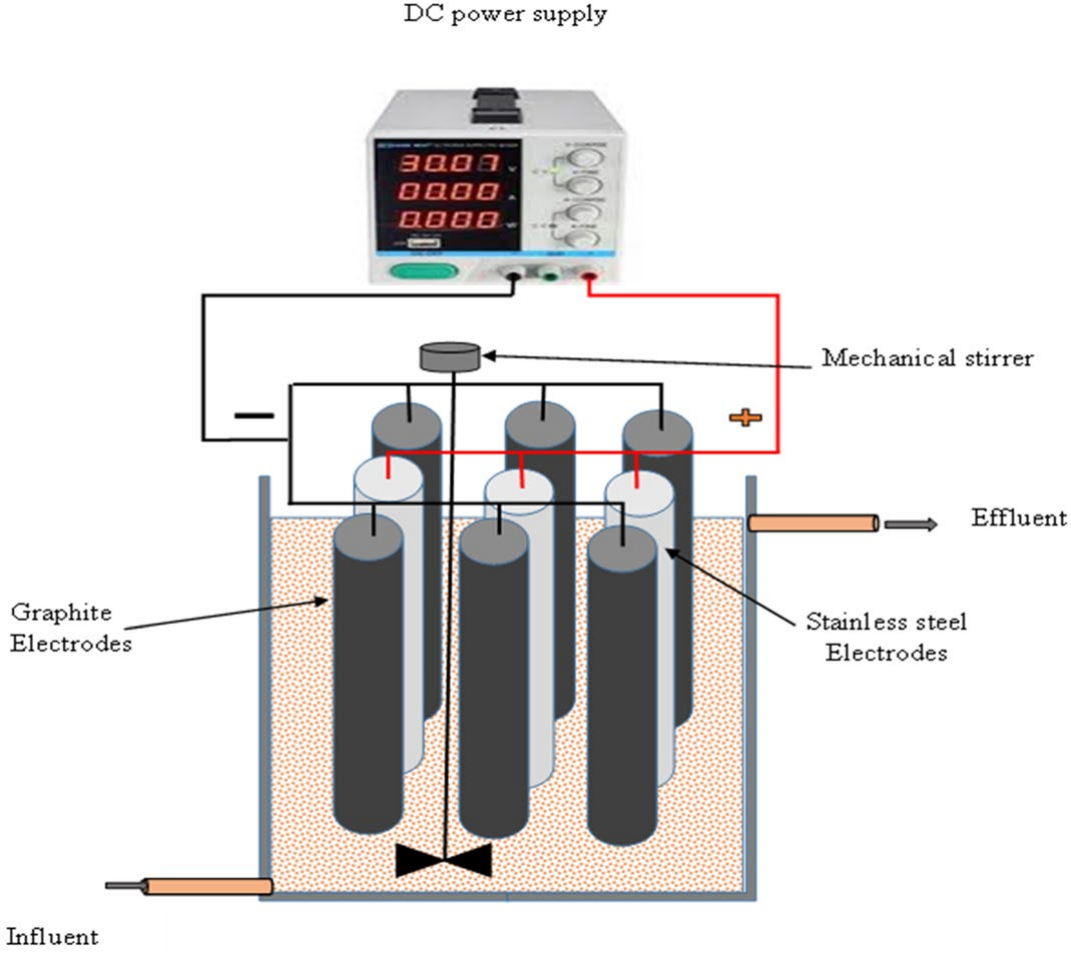
Electrooxidation treatment cell.

In this study, the experimental setup is given schematically in Fig. 1. Electro-disinfection treatment was done in an electro-oxidation cell in an electrolytic cell designed based on patent No. EGPO 30235^16^ is made of a Perspex glass container with a net volume of 3 L. and fabricated of a Perspex glass material container with a net volume of 3 L. The EC unit has dimensions of 12.5 cm × 12 cm × 20 cm (L × W × H), while the water level was kept below 5 cm resulting in an adequate volume of 2.25 L. Nine identical cylindrical shape electrodes were distributed in three horizontal, and two rows have 6 graphite electrodes between them; one contains three stainless electrodes with a binary distance between all electrodes of 2 cm. Each electrode has a 15 cm height, a 1.6 cm diameter, and a volume of about 12 cm^3^ and the working surface area is 0.054 m^2^. Accordingly, the total volume of the electrodes in the cell is 0.24 L, which means that the adequate volume of the cell is about 2 L. The electrodes were connected to the D.C power supply with a variable output voltage range from 0 to 30 V and current from 0 to 10 A. The treatment runs were performed at constant temperature (25 °C), mixing speed (250 rpm), and with 2 L of synthetic contaminated solution.

### Operating conditions

The study is conducted in several experimental runs; each one represents operational parameters such as contact time, conductivity, microorganisms count, and current density. The effect of operational time was investigated by applying the current density of 2 mA/cm^2^ at TDS 1000 mg/L for 30 min. The current density was investigated for values of 2, 4, 6, and 8 mA/cm^2^ at the determined effective time. The effect of electrolyte concentration was analyzed by preparing the electrolyte solution of sodium chloride (NaCl) with the concentration of 17.5, 35, 52.5, 70, and 87.5 mM to obtain TDS values of 1000, 2000, 3000, 4000, and 5000 mg/L while different biomass and bacterial densities were evaluated at the received effective time, TDS and current density. In each experiment, treated samples were collected in sterile tubes; bacterial concentration was evaluated for treated and control samples. Before each run, the electrodes were washed to remove any surface attachments and impurities on electrodes with a solution freshly prepared by mixing 100 mL of HCl solution (35%) and 200 mL of chloroform solution. After the electrodes were washed, they were dried in the oven at 105 °C to remove the residuals on their surfaces of bacterial contamination.

### Wastewater sources

#### Simulated wastewater

This study used simulated wastewater containing non-pathogenic *E. coli* as a model microorganism. The bacterial stock culture was obtained by seeding some pure colonies in a sterile nutrient broth medium (5 mL) incubated at 37 °C for 24 h. Fresh Hektoen medium (500 mL) was used to inoculate 3% (v/v) of this culture for two incubation times (24 h/each at 37 °C). Before any experiment, a certain amount of *E. coli* culture (100 mL) was centrifuged at 6000 rpm for 10 min. The obtained biomass was washed twice with sterile standard saline solution (9 g/L NaCl). After centrifugation, it was subsequently resuspended in the sterile solution of distilled water and sodium chloride with different concentrations to achieve a final concentration of around 10^6^–10^7^ CFU/mL.

#### Real wastewater

After obtaining the best-operating conditions, two real wastewater sources were collected and subjected to an electrooxidation unit. The first source is the agricultural drain in Menufia governorate that receive agricultural drainage water mixed with sewage. The other source is the effluent from the secondary treatment wastewater treatment plant at Giza, Egypt. prior chlorine tank. The wastewater was fed continuously using a peristaltic pump under adequate conditions.

### Wastewater analysis

Bacterial indicators, pH, and NaCl were analyzed for each experimental run. To evaluate the bacterial concentration in each experiment after the treatment of the water sample (level of removal of *E. coli* by electrochemical disinfection), influent and effluent samples were collected in sterile tubes. Influent samples were serially diluted in a standard saline solution. Diluted and undiluted samples (1.0 mL) were seeded in triplicate on nutrient agar plates and incubated at 37 °C for 24 h.

For real wastewater samples, complete analysis was characterized at the best applicable conditions in terms of pH, (COD), biological oxygen demand (BOD), total suspended solids (TSS), total Kjeldahl nitrogen (TKN), ammonia nitrogen (NH_4_–N), nitrite (${\mathrm{NO}}_{2}^{-}$). nitrate (${\mathrm{NO}}_{3}^{-}$), oil and grease total phosphorus and trihalomethanes beside the bacterial indicators heterotrophic bacteria, total coliform, fecal coliform (thermophilic), *Streptococcus faecalis,* and *Pseudomonas aeruginosa.* Heterotrophic bacteria were measured in the R2A agar medium by counting colonies formed for each plate (colony-forming unit (CFU)). These analyses, unless otherwise specified, were performed according to the Standard Methods for the Examination of Water and Wastewater^17^.

### Calculation of removal efficiency, operating cost, and energy consumption

The percentage removal efficiency r was calculated using Eq. (1):

1 $$\% \;{\text{Removal}} = \frac{{{\text{C}}_{0} - {\text{C}}}}{{{\text{C}}_{0} }} - 100$$

where C_0_ is the initial concentration and C is the final concentration of the pollutant.

The operating cost is one of the most critical parameters in the EC process because it affects the application of any wastewater treatment method. The operating price includes material (mainly electrodes), electrical energy, labor, maintenance, and other expenses. The latter cost items are mainly independent of the electrode material. Thus, this study calculated the operating cost as electrical energy costs. The energy consumption is consumption quantities per m^3^ of wastewater treated. The calculation of energy consumption is expressed as in Eq. (2)

2 $$\text{E}_{C}=\frac{(\mathrm{V} x \mathrm{I}x \mathrm{t})}{\mathrm{v}}$$

where E_*C*_ is energy consumption (kWh/m^3^), V is voltage (Volt), I is current (Ampere), t is contact time (seconds), and v is the volume of the treated wastewater (m^3^), respectively.

## Results and discussions

### Electro-disinfection of synthetic microbial contaminated water

The bacteria *E. coli* was selected as a model of pathogens for preparing synthetic wastewater in the disinfection study due to its high environmental and sanitary risks. The electro-disinfection of synthetic water contaminated with the *E. coli* bacteria was studied in terms of operational characteristics such as the concentration of *E. coli* as a function of the experimental time of exposure, current density, TDS, and microbial cell densities.

#### Effect of electro-disinfection contact time

**Figure 2:**
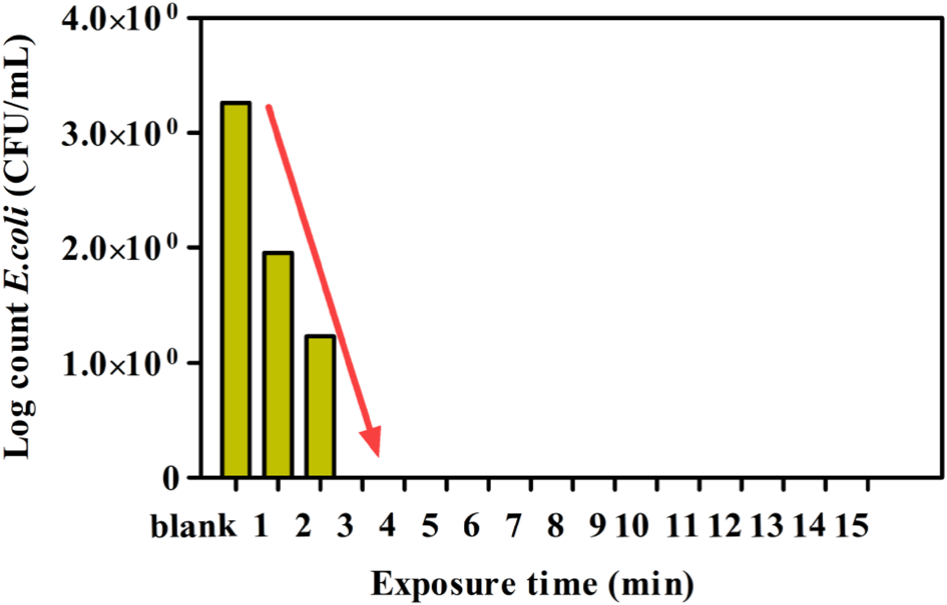
Effect of exposure time (1–15 min) on *E. coli* during electrochemical inactivation treatment. Experimental data are shown as mean ± standard deviation (n = 3).

The effect contact time experiment was performed at the current density of 4 mA/cm^2^ and electrolyte concentration of 17.5 mM NaCl (1000 mg/L) with the initial *E. coli* log count of 3.2 CFU/mL. Samples were collected in sterile tubes every min, starting from zero to 15 min. The results in Fig. 2 show that a 100% *E. coli* elimination occurred at the third min of contact time. *E. coli* count was reduced to 1.2 and 1.9 CFU/mL after 1 min and 2 min exposure times, indicating that disinfection performance of more than 99.98% required a residence time of 2 min. The obtained time is auspicious, cost-effective, and better than other electro-disinfection studies carried out by Herraiz-Carbon et al.^18^, who studied disinfection of polymicrobial urines from hospital wastewater using electro-oxidation units. They achieved 4–6 logs of bacterial removal after 180 min using the electrooxidation reactor with two boron-doped diamond and stainless-steel electrodes at the current density of 50 A/m^2^. Correspondingly, the results proved that electro-oxidation is significantly more effective than chemical disinfection using chlorine, where chlorination requires longer exposure (at least 30 min) to achieve a bactericidal performance of 99.94% or higher^19^. Time is essential in designing a disinfection technique related to power and chemical consumption and capital construction^13^. Accordingly, the other operating parameters were studied as a function of time to determine their optimal values.

#### Effect of current density

**Figure 3:**
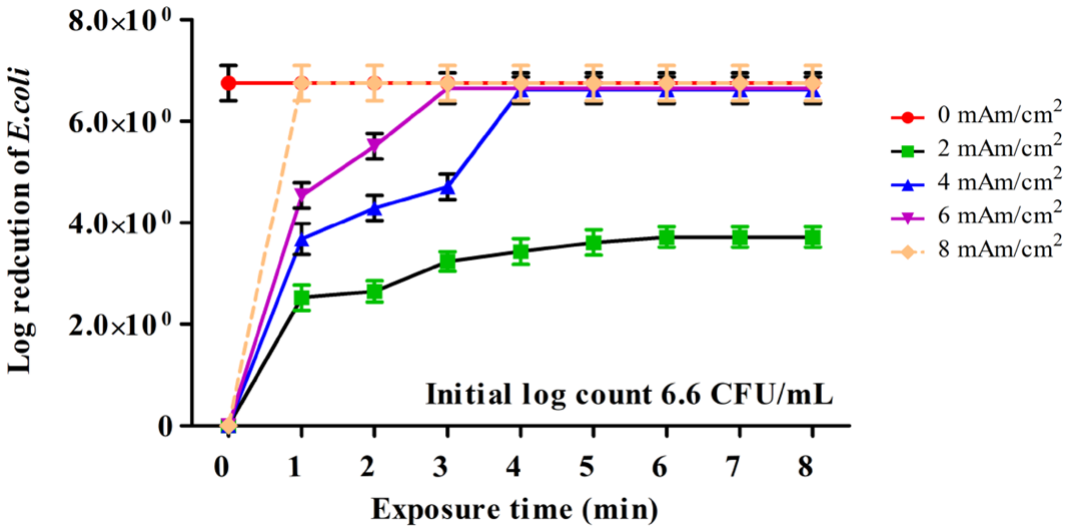
Effect of different current densities (2–8 mAm/cm^2^) on *E. coli* bacterial inactivation during electrochemical inactivation treatment of solutions containing initial concentration of ∼ 10^6^ CFU/mL *E. coli*. Experimental data are shown as mean value ± standard deviation (n = 3).

The current density is a significant factor as it determines the power consumption cost and electrode dimensions. The effect of current density as a function of time on the electro-disinfection was carried out at the electrolyte concentration of 17.5 mM NaCl (1000 mg/L) and doubled initial *E. coli* log count (6.6 CFU/mL) with different current densities 2, 4, 6, 8 mA/cm^2^. Figure 3 shows the log reduction of *E. coli* at different current densities. Application of 2 mA/cm^2^ was not very efficient for the removal of *E. coli,* and the maximum log counts reduced was about 3.7 CFU/mL at 6 min. The reduction rate was fixed after this time, indicating that more current is required to remove high logs values. Increasing current density by two folds to 4 mA/cm^2^ showed a gradual reduction of *E. coli* starting with a log reduction of 3.9 CFU/mL at the first min, reaching complete removal at the fourth min. The same behavior was observed at 6 mA/cm^2^ as the gradual reduction was observed in the third min, at which complete removal was achieved. The entire reduction of *E. coli* of cell densities was performed within the first min at 8 mA/cm^2^, indicating that the contact time could be seconds at that current density.

The results are consistent with those of Ghasemian et al.^20^, who proved that increasing the current density from 0.2 to 1 mA/cm^2^ led to a more than 50% reduction in log10-bacterial concentration after 20 min, and within 5 min for 2 mA/cm^2^, 2 min for 4 mA/cm^2^ using the electrochemical unit with doped tin-tungsten-oxide electrodes. Increasing the applied current density resulted in enhanced inactivation efficacy of *E. coli*. The main reason for this phenomenon is that increasing current densities accelerate the movement of the electrons, which increases the generation of more oxidizing species at the anode surface, which attack and destroy bacteria^21^.

Furthermore, a tremendous amount of electrical charge will pass through the electrolyte with a more significant current density, potentially resulting in faster disinfection rates^22^. However, from the economic point of view, the high current density could not be cost-effective due to the high consumption rate of electrical power. Accordingly, 4 mA/cm^2^ was chosen as the effective current density.

#### Effect of different electrolyte concentrations

**Figure 4:**
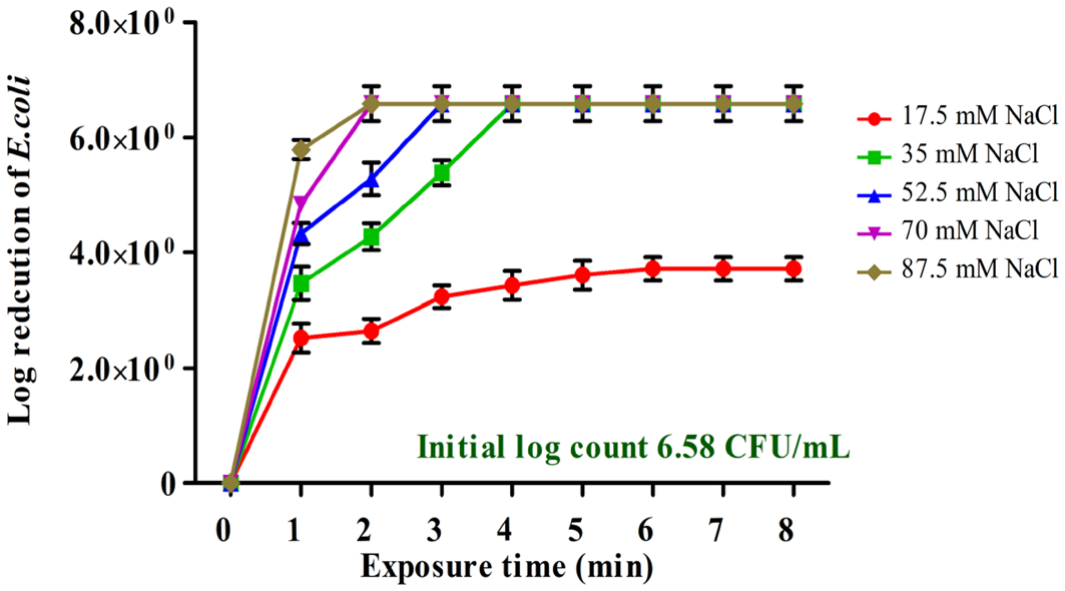
Effect of different concentrations of electrolyte concentration (17.5–87.5 mM NaCl) on *E. coli* during electrochemical inactivation treatment Experimental data are shown as mean ± standard deviation (n = 3).

Synthetic wastewater was prepared with different electrolyte concentrations to obtain values of about 17.5, 35, 52.5, 70, and 87.5 mM NaCl, corresponding TDS values from 1000 to 5000 mg/L. The experiment was carried out at a current density of 2 mA/cm^2^ and time of up to 10 min. The results in Fig. 4 showed that TDS strongly affects the removal of *E. coli* with electro-disinfection. The increase of TDS concentration in the electrolyte solution is accompanied by a high reduction of *E. coli* in a shorter time. The electrolyte concentration of 17.5 mM NaCl (1000 mg/L) is insufficient for the complete removal of *E. coli* as the maximum log reduction achieved was about 5.5 CFU/mL after 6 min contact time. The initial log count of *E. coli* dropped gradually over 4 min in a solution of 35 mM NaCl (2000 mg/L), while 3 min was enough to eliminate *E. coli* at 52.5 mM NaCl (TDS 3000 mg/L).

In contrast, complete disinfection was achieved in a shorter time (2 min) for 70, and 87.5 mM NaCl (4000 and 5000 mg/L) as a whole log decrease (6.5 CFU/mL) was achieved with a current density of 4 mA/cm^2^. The performance of electro-disinfection has worsened at low TDS concentrations, which suggests at the respective current level of 2 mA/cm^2^, TDS concentrations of 2000 and 3000 mg/L were found to be sufficient for effective inactivation of *E. coli* in this study. The results were directly compared with the previously reported findings by Ghasemian et al.^20^, where the complete disinfection of *E. coli* in 100 mM NaCl was obtained at 2 min with the current density of 4 mA/cm^2^, respectively.

#### Effect of cell densities of *E. coli*

**Figure 5:**
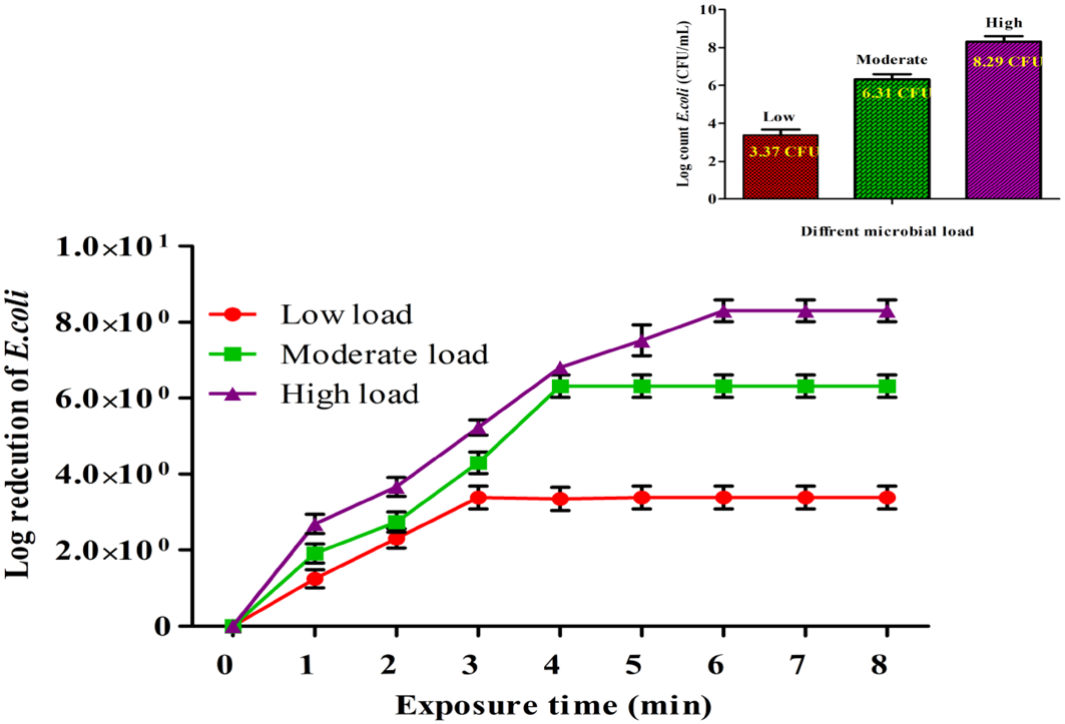
Effect of various bacterial populations on *E. coli* during electrochemical inactivation treatment. Experimental data are shown as mean ± standard deviation (n = 3).

From the previously obtained results above, the best condition for electro-disinfection was a TDS concentration of 2000 mg/L (35 mM NaCl), a current density of 4 mA/cm^2^, and a contact time of up to 5 min. At these conditions, three bacterial solutions of *E. coli* with different densities (Log count 3.37 as a low, 6.31 as a moderate, 6.31, and 8.29 as an intense, high CFU/mL) were prepared and subjected to electro-disinfection. The inactivation of *E. coli* cells is perceived in Fig. 5. In truth, bacterial damage is evident at low cellular density. The results revealed that the low bacterial load (log 3.37 CFU) was destroyed within 3 min of treatment, and *E. coli* cell survival was substantially and quickly reduced. At higher bacterial cell densities, it was found that log 6.31 and log 8.29 CFU/mL of *E. coli* were effectively inactivated after 4 min and 6 min, respectively. The findings align with previous research that shows that low densities of *E. coli* cells might be easily destroyed in a shorter amount of time following inactivation^23^. In this study, the EC inactivation, chlorination, ozonation, and the Fenton reaction were all found to be successful in eliminating *E. coli* with a starting density of 10^8^/mL from wastewater in their investigation. With an elimination rate of 99.4% or better, nearly all of the cells in the disinfected samples lost their viability in terms of being physiologically available for incubation^24^.

### Proposed mechanism of *E. coli* elimination using electro-oxidation

The formation of powerful oxidants like oxygen, ozone, or hypochlorite in the anode electrode throughout the electrolysis of water serves as the major driving force behind the electro-disinfection process employing the electro-oxidation unit. These chemical oxidants are produced when plunging electrodes apply electric current to aqueous microbe solutions. When the solution contains salts such as chloride salts, electrolysis has a range of oxidants, including hydrogen peroxide and ozone when oxygen is available, as well as free chlorine and chlorine dioxide when chloride ions are present^25^. However, the type of oxidant depends on the applied current, electrolyte solution, and anode electrode type^26^. For example, Anodes used for the process of electro-disinfection by hypochlorite ions should have a low overpotential toward chlorine gas evolution, such as platinum electrodes. However, pure Pt anodes are not used in industrial applications because of their high costs, and the alternatives for Cl_2_ gas evolution are PbO_2_ electrodes^27^.

Electro-disinfection by oxygen gas resulting from the anodic generation of oxygen, which shows some germicidal activity, is used mainly for removing microorganisms from water in small applications where the generation of chlorine species is undesirable. The most used electrodes for oxygen evolution are stainless steel and graphite electrodes.

**Figure 6:**
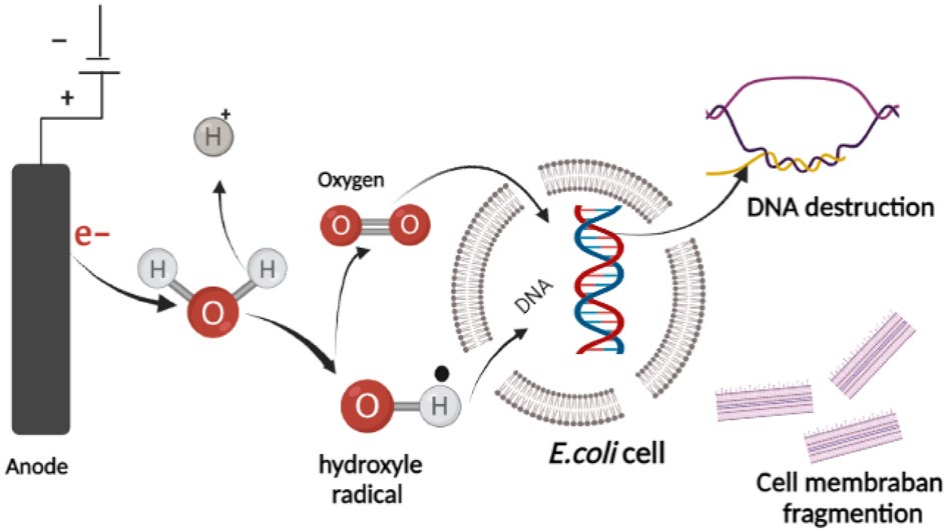
Proposed scheme of *E. coli* disinfection in the electrooxidation cell.

For our study, the removal mechanism is probably the destruction of *E. coli* due to the production of oxidants derivatives of oxygen caused by the applied electric field, which causes irreversible permeabilization of cell membranes^28^. The type of electrodes used in this study are graphite at the anode and stainless steel at the cathode; with the short contact time, it was not possible to generate choline compounds for disinfection. The scheme of the proposed electro-disinfection process is illustrated in Fig. 6.

As can be seen in Fig. 6, disinfection of pathogens occurred due to oxidation by OH^⋅^ radical species produced from the oxidation of water at the anode; the inactivation process takes place within the surrounding area of the electrode and solution interphase. Therefore, as indicated in Eq. (3), powerful oxidant species are generated from electrolyte electrooxidation at the anode surface, resulting in the direct oxidation process of pathogen^29,30^. Hence, depending on the current density applied, the oxidation of pathogens is identified to occur through direct electron transfer in the potential region before oxygen evolution via electrogenerated OH^⋅^^31^. In addition, the water oxidation reaction for the generation of OH^⋅^ is always in competition with the secondary reaction of anodic dissociation of these radicals in oxygen and with the oxygen evolution reaction as indicated in Eqs. (4) and (5)^32^.

3 $${\text{M }} + {\text{ H}}_{{2}} {\text{O}} \to {\text{M}}^{ \cdot } \left( {{\text{OH}}} \right)^{ } + {\text{H}}^{ + } + {\text{e}}^{ - }$$

4 $$2{\text{OH}}^{ \cdot } \to {\text{O}}_{{2}} + {\text{2H}}^{ + } + {\text{2e}}^{ - }$$

5 $$2{\text{H}}_{{2}} {\text{O}} \to {\text{O}}_{{2}} + {\text{4H}}^{ + } + {\text{2e}}^{ - }$$

As the OH^⋅^ radical species are considered the main disinfectant agent of pathogens in the electro-oxidation process owing to their high oxidation potential 33, studies proved a direct relationship between its anode’s surface and the radical activity^15^. Accordingly, graphite as the non-active anode is favorable due to its high oxygen over-potential, achieving the oxidation of pathogens by an electrochemical step mediated by physisorbed OH radicals.

**Figure 7:**
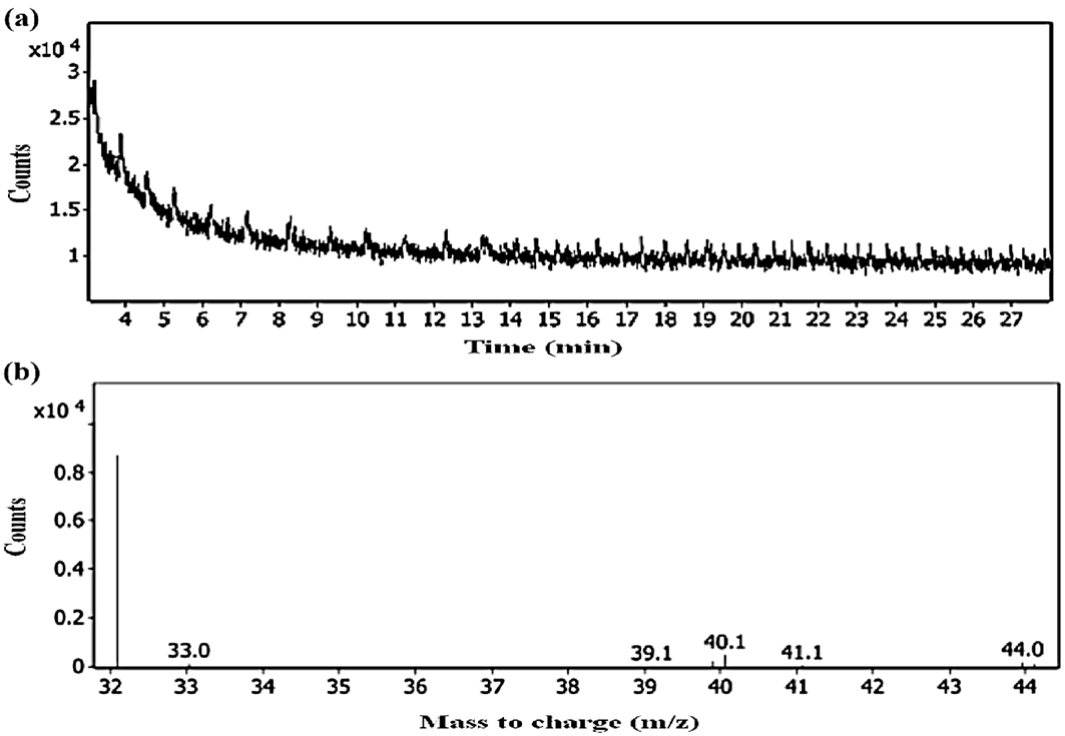
Gas chromatography for the solution after disinfection (**a**) scan of chlorinated compounds vs. time (**b**) mass to charge spectrum.

This proposed inactivation pathway was also confirmed with a gas chromatographic analysis of the solution after disinfection with electro-oxidation at these conditions for detecting any chlorinated by-products (DBPs) as a result of the generation of chlorine compounds (Fig. 7) and free chlorine determination in the solution. The results showed no detection of any halogenated or chlorinated compound in the solution. Accordingly, the mechanism of inactivation of *E. coli* in this study is the electro-disinfection by oxygen gas.

### Application of electro-disinfection on real wastewater under continuous operation

The results obtained in the previous section indicated that electro-disinfection could be applied at retention times required for continuous mode operation, calculated from the batch mode operating experiments, and two different operating flow rates of 360 mL/min for 5 min 600 mL/min for 3 min were elected. Under these conditions, wastewater from the secondary wastewater treatment plant and agricultural drainage was subjected to electro-disinfection continuously to simulate a full-scale electro-oxidation treatment cell. The treated wastewater was collected every min to investigate the best time for disinfection for each type of wastewater.

#### Application of drainage wastewater

**Figure 8:**
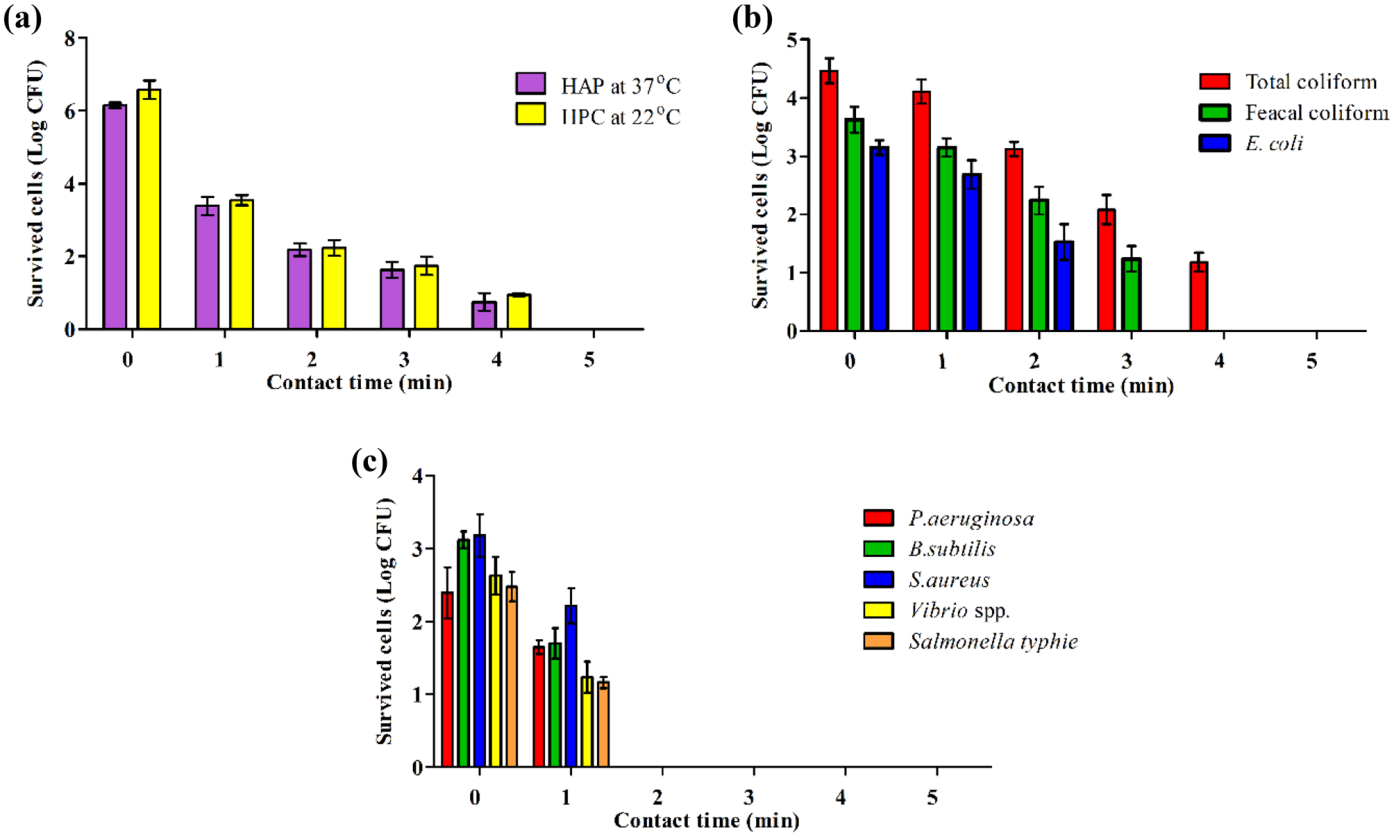
Efficacy of electrochemical disinfection, as a function of time, of some waterborne bacterial species (**a**) HPC at 37 °C and 22 °C, (**b**) fecal bacterial indicator, (**c**) some potential pathogenic bacteria for drainage wastewater under stable conditions.

The raw drainage wastewater was subjected to disinfection by applying the current density of 4 mA/cm^2^, TDS of 987 mg/L and retention times required for continuous mode operation were calculated from the batch mode operating experiments, 5 min and operating flow rate of 360 mL/min. At the start of the experiment, samples were collected from the effluent at zero time and every min till 5 min, representing the actual retention time. The results depicted in Fig. 8 indicated that the bacterial densities of total heterotrophic bacteria, coliform group (TC, FC, and *E. coli*), and some pathogenic species, including *P. aeruginosa*, *B. subtilis, Vibrio* spp., and *S. typhie* was, initially enumerated in all samples before and after disinfecting with ED within varying exposure time. The average log counts as 6.2, 6.6, 4.6, 3.6, 3.2, 2.3, 3.13, 3.17, 2.6, and 2.2 CFU/mL, respectively for HPC at 37 °C, HPC at 22 °C, TC, FC, *E. coli, P.aeruginosa*, *B. subtilis, Vibrio* spp., and *S. typhie*. The results showed that *E. coli* and pathogenic bacteria were completely eradicated after 3 min exposure time. This result is in agreement with the obtained results from batch experiments on synthetic wastewater under the same conditions. As the TDS concentration is about 1000 mg/L, the whole electro-disinfection time was about 5 min, with slight residuals of total bacterial counts still existing. In addition, the high bacterial load in the wastewater affected the time required for the disinfection. Likewise, the sample was collected during continuous experiments and subjected to physico-chemical characterization, as depicted in Table 1. The results showed slight removal of chemical pollutants, which is anticipated due to the short treatment time. Similarly, there was no detection of chlorinated organic compounds such as THMs, indicating no formation of chlorin derivatives as a result of electrolysis.

**Table 1:** Physico-chemical characterization of agricultural drainage water before and after electro-disinfection.

Parameters	Units	Result	
		Before	After
Flow rate	mL/min	360	
HRT	min	5	
Operation time	min	30 min	
pH	–	7.14	7.35
Total dissolved solids	mg/L	987 ± 25	1071 ± 30
Total suspended solids	mg/L	15 ± 0.45	10.5 ± 0.5
Chemical oxygen demand	mgO_2_/L	36 ± 2	25 ± 1.7
Biochemical oxygen demand	mgO_2_/L	12 ± 1.1	9 ± 0.9
Ammonia	mg/L	3 ± 0.23	< 0.1
Total sulfides	mg/L	2 ± 0.1	< 0.1
Oil and grease	mg/L	8 ± 0.55	2 ± 0.3
Tri-halomethane	µg/L	–	Nill

From the outcomes mentioned above, it could be concluded that the utilization of lower current density may avoid higher power consumption for the disinfection process. The complete inactivation of *E. coli* was attained with 5 min contact time, 4 mA/cm^2^ of current density, and 1000 mg/L of TDS concentration. These optimized conditions would be involved in the disinfection strategy in wastewater treatment steps.

#### Application of secondary treated municipal wastewater

**Figure 9:**
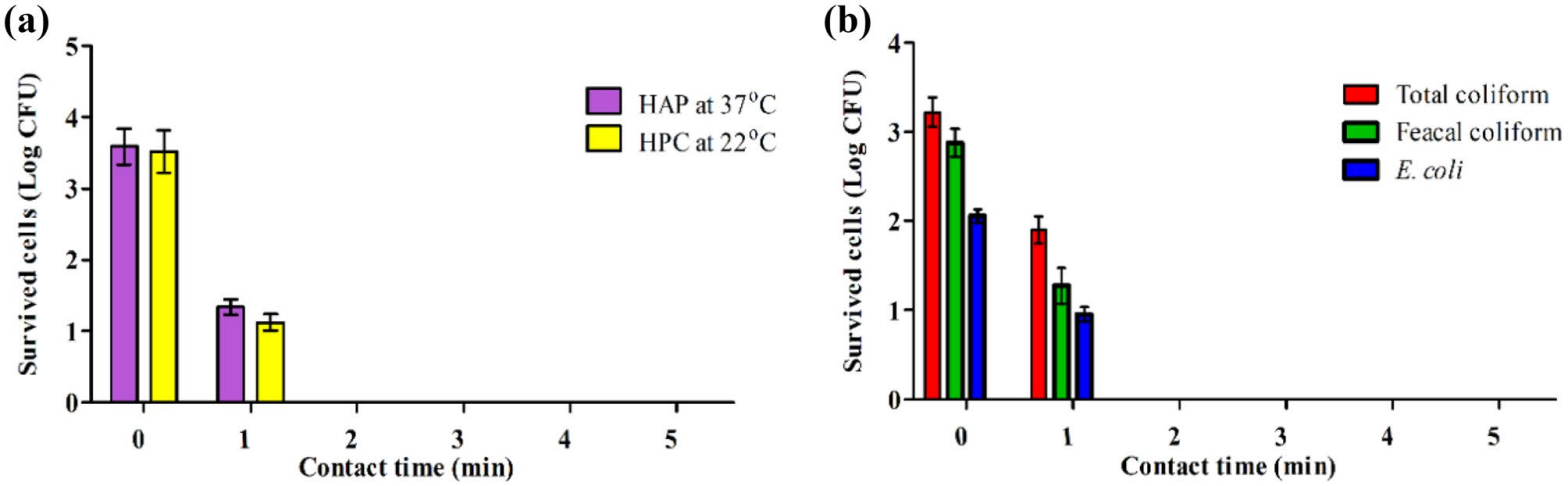
Efficacy of electrochemical disinfection, as a function of time, of some waterborne bacterial species (**a**) HPC at 37 °C and 22 °C, (**b**) fecal bacterial indicator for drainage wastewater under stable conditions.

Due to the excessive microbial burdens inherent in wastewater effluents, disinfection operations for effluents have become necessary to improve wastewater treatment facilities' performance and eradicate dangerous bacteria^33^. Within wastewater, a vast range of pathogenic organisms survive, but if not effectively managed, they can cause a significant threat to public health^34^. During the ultimate effluent release, wastewater effluents may transport harmful microbes into watersheds. Some dangerous diseases, such as cholera, typhoid, and hepatitis A may occur due to polluted rivers. Polluted rivers, notably seafood, cockles, and shrimp, might damage sea life. Those who consume this poisoned seafood risk getting extremely unhealthy^35^. Therefore, the secondary treated wastewater was subjected to continuous electro-disinfection for drainage wastewater. The experiment was carried out at a current density of 4 mA/cm^2^, the TDS was found to be 1987 mg/L, and the hydraulic retention time was adjusted to 3 min. The samples were collected every minute, starting from zero to 5 min, to investigate the time of electro-disinfection. The results depicted in Table 2 and Fig. 9. showed the complete removal of all bacterial indicators after the first min. Monitoring the bacterial densities of HPC at 37 °C, HPC at 22 °C, TC, FC, *E. coli*, *P. aeruginosa, B. subtilis, Vibrio* spp*.,* and *S. typhie* showed that a comprehensive decline in the number of bacteria occurred after 2 min of exposure time. Although the secondary treated municipal wastewater sample contained a large proportion of COD, mor than 50% of this organic matter was removed as result of electro-oxidation. Besides, the examination of the presence of halogenated organic compounds indicated that no formation of these compound during the disinfection process.

**Table 2:** Physico-chemical characterization of secondary wastewater before and after electro-disinfection.

Parameters	Units	Result	
		Before	After
Flow rate	mL/min	600	
HRT	min	3	
Operation time	min	30 min	
pH	–	7.1	7.54
Total dissolved solids	mg/L	1997 ± 29	2080 ± 38
Total suspended solids	mg/L	15 ± 2	10.5 ± 1.2
Chemical oxygen demand	mgO_2_/L	52 ± 3.3	23 ± 0.9
Biochemical oxygen demand	mgO_2_/L	21 ± 1.5	11 ± 0.4
Ammonia	mg/L	3.2 ± 0.1	< 0.1
Total sulfides	mg/L	2 ± 0.23	< 0.1
Oil and grease	mg/L	9 ± 0.8	2.5 ± 0.2
Tri-halomethane	µg/L	–	Not detected

Electrochemical disinfection, analogous to standard chemical disinfection strategies, would indeed happen after clarification system and filtration processes in water purification^36^. Hence, after secondary treatment, the wastewater sample was selected to be disinfected with electrooxidation. It is well-known that the microbial population densities in secondary treated wastewater are lower than in untreated or raw wastewater, as the treatment process could efficiently reduce the microbial load during the operational process. Up to 99% of fecal indicator bacteria (FIB) can be dismissed using primary and secondary treatment processes^37^. According to the FIB levels in the influent, this level of reduction may not be adequate to fulfill the standard of specifications for using handled domestic wastewater for agriculture and irrigation uses and enabling leisure activities in receiving water bodies^38^.

### Techno-economic evaluation

Electro-disinfection in this study involved creating a direct current (DC) by graphite anode and stainless steel cathode that pass through the water and prohibit the growth of microbes. The results obtained showed some technical benefits, including ease of use and installation, simple equipment, and high-performance treatment effectiveness^39^. requirement less energy, making it affordable and ecologically friendly technology, and it might be operated using fuel batteries or solar system^9^. As a result, the inactivation effectiveness of *E. coli* was studied using a variety of process factors, including the. The optimized settings were utilized to disinfect real wastewater samples.

The effective operating conditions for this study are pH 7–8, TDS > 2000, the current density of 4 mA/cm^2^, and the operation time of 3–5 min. At these conditions, real wastewater samples were adjusted to continuous flow in the reactor through feeding with a peristaltic pump to simulate the actual application of such treatment technology. After the continuous experimental study on the different operating parameters, the operational cost was calculated based on the operation time and electrical consumption. Data represented in Table 3 displayed the complete elimination of the initial log count (3.2 CFU/mL) of *E. coli* in real drainage wastewater after 3 min of operation. Further, the results revealed this study's cost estimation and power consumption. Based on the commercial prices in Egypt, the cost of electricity for commercial use is L.E 1.6 /kWh (about 0.09 $/kWh). The cost of sodium chloride added to the solution to increasing the TDS to 2000 mg/L was calculated based on the amount required, which was 1.2 kg/m^3^, and the cost of commercial salt was 0.25 L.E/kg. Accordingly the cost of added sodium chloride salt is 0.3 L.E/m^3^ (0.15$/m^3^) the operation cost at 4 mA/cm^2^ ranged from 0.57 L.E/ m^3^ (0.03 $/m^3^) to 1.7 L.E/m^3^ (0. 89 $/m^3^). The operating cost at the operating conditions (TDS 2000, 4 mA/cm^2^ and 3 min) were calculated as 1.13 L.E/ m^3^ (0.06 $/m^3^). This cost is meager compared to conventional disinfection methods that could achieve the same efficiency. From these calculations, electro-disinfection is a promising solution for application in wastewater treatment plants because of its modular design, high efficiency, and ease of automation and transportation. This study looked at three different scenarios: water treatment, centralized wastewater treatment, and distributed wastewater treatment.

**Table 3:** Electrical consumption and cost estimation at best-operating conditions.

Time (min)	Current density	Log removal of *E. coli*	Electrical consumption (kWh/m^3^)	Total running cost ($/m^3^)
0	4 mA/cm^2^	0	0.0	0.0
1		0.8	0.2	0.030
2		1.4	0.3	0.045
3		3.2	0.5	0.060
4		3.2	0.7	0.074
5		3.2	0.9	0.089

## Conclusion

In the current investigation, the bacterial indicator's electro-disinfection relied on current density, time, and TDS concentration. *E. coli* was found to completely remove after 5 min time TDS 1000 mg/L and 4 mA/cm^2^ current density at initial *E. coli* log count of 6.6 CFU/ml. The increase in current density and TDS concentration decreased the time required for disinfection. However, from the economic point of view, this might increase operating costs due to high power consumption and chemical added to increase the TDS. The electro-oxidation unit configuration with the order of the electrodes had a determining impact on the reactor performance, where the configuration and types of cathode and anode direct the type of electro disinfection to use oxygen gas to carry out the disinfection process. The added chloride concentrations did not significantly affect free chlorine production in the reactor. Cost analysis for disinfection of secondary wastewater treatment effluent indicated that graphite and stainless steel electrodes in the electro-disinfection unit are more appropriate due to lower power consumption (0.5 kWh/m^3^) and lower operating cost (0.06 $/m^3^) compared to conventional methods.

## Data Availability

The datasets used and/or analyzed during the current study are available from the corresponding author upon reasonable request.
